# A qualitative study of career decision making among African and Asian international medical students in China: process, challenges, and strategies

**DOI:** 10.1007/s10459-024-10329-z

**Published:** 2024-04-09

**Authors:** Wen Li, Hong Sun, Asaduzzaman Khan, Robyn Gillies

**Affiliations:** 1 School of Education, Faculty of Humanities and Social Sciences, The University of Queensland, Brisbane, QLD 4072 Australia; 2 School of Basic Medicine, Xuzhou Medical University, Xuzhou, 221004 China; 3 School of Health and Rehabilitation Sciences, Faculty of Health and Behavioural Sciences, The University of Queensland, Brisbane, QLD 4072 Australia

**Keywords:** Africa, Asia, Career decision making, Cognitive information processing, International medical student, Career guidance

## Abstract

China hosts around 68,000 international medical students (IMSs) primarily from lower income countries in Africa and Asia, who have the potential to contribute to international medical services. Understanding how these IMSs make career decisions can help better address the issue of global medical workforce shortage. However, such research is limited. Our study aims to explore the career decision-making process of China-educated IMSs, the challenges they experienced and the strategies they employed.

In this exploratory qualitative study, we conducted semi-structured interviews with IMSs educated in China in 2022 using purposeful sampling. Twenty virtual one-on-one interviews were conducted, and data were analysed through directed qualitative content analysis. Cognitive Information Processing (CIP) theory was applied as the guiding framework for organising and analysing the data.

The career decision-making process of the participants generally followed the stages of decision-making cycle in CIP theory, with a combination of urgent migration decisions and specialisation considerations adding layers of complexity to their career trajectories. Identified challenges encompassed lack of knowledge about oneself and career options, lack of decision-making skills, concerns of contextual complexities that limited the career decision-making process, low motivation and negative thoughts. Specific challenges due to their role as IMSs arose, which were related to career information access, self-capability evaluation, degree accreditation, employment competitiveness and mental states. Participants’ proposed strategies were categorised into personal and institutional aspects, providing insights into addressing these challenges.

This study substantiates and expands the application of the CIP theory within the sphere of the particular cultural and educational context of IMSs educated in China. It highlights the significance of integrating migration decision-making into career guidance for IMSs, and contributes to the literature by proposing an evidence-based tiered career intervention programme for IMSs.

**Supplementary Information:**

The online version contains supplementary material available at 10.1007/s10459-024-10329-z.

## Introduction

It is not a new story that some western countries host international students for medical studies, under diplomatic collaboration schemes (Chur-Hansen, 2004; Patel & Araya, 1992) or due to financial benefits considerations (Breen & Birrell, 2020; Wilkinson, 2022). Documents suggests that annually over 600 international medical students (IMSs) enrol at medical schools in Australia (Breen & Birrell, 2020), and around 750 IMSs enrol in the UK (Knight, 2019; Office for Students, 2021). As the trend of internationalisation of medical education prevails, some countries have newly emerged as popular destinations for IMSs through creating English-parallel medical programmes that have been targeted at foreign citizens during the past two decades (Mayberry, 2013; OECD, 2019). Each year, approximately 1,700 IMSs and 1,900 IMSs are admitted in English-medium medical programmes in Romania and Poland respectively, with the regional recognition of medical qualifications and active marketing strategies facilitating the enrolment volume (OECD, 2019). Similarly, international medical education is also thriving in low- and middle-income countries (LMICs) in recent years, and the number of IMSs in some LMICs, despite the relatively short history, is surprisingly high and has exceeded that in high-income countries (HICs) (Li & Sun, 2019; Mospan & Slipchuk, 2020).

Around 68,000 IMSs are currently studying in China’s universities (Li et al., 2023), averaging an annual intake of more than 10,000 IMSs across the country (Mayberry, 2016), among whom approximately 90% are self-financing (Jiang et al., 2022). Unlike HICs, where IMSs consist of a mixture of citizens with various nationalities (Breen & Birrell, 2020; OECD, 2019), China attracts IMSs overwhelmingly from lower economies in Africa and Asia (Li & Sun, 2019). These students choose to study medicine in China due to reasons such as the failure to secure a seat in the fiercely competitive medical programmes in their home countries, the absence of medical schools in their home countries, the preference of lower tuition fee, the English medium of instruction, or the intention to obtain an overseas study experience in a country with better standards of technology in healthcare (Anjali et al., 2016; Bernard et al., 2021; Jiang et al., 2022).

Different from IMSs studying in western countries, the majority of whom attempt to stay there for medical practice (Datta & Miller, 2012; McGrail et al., 2019; OECD, 2019), most IMSs studying in China only receive their education in Chinese medical schools before returning to their home country or moving forward to another foreign country to build their medical careers (Li & Sun, 2019; Raza, 2014). In the year of 2022 alone, 2064 China-educated Indian medical graduates passed the Foreign Medical Graduates Examination and were qualified to work as a doctor in India, accounting for 17.0% of the total qualified candidates who took this exam that year (National Board of Examinations in Medical Sciences, 2022). The UK, a popular migration destination for China-educated IMSs (Li & Sun, 2019), had 502 registered doctors who graduated from English-language medical programmes in China as of February 2022 (Rashid et al., 2023). Given the significant number of IMSs in China and the potential they have in supplying the medical labour market in their home countries as well as other countries, it is of vital importance to understand their career decision making, as their career decisions may contribute to alleviating the physician shortage and health inequity in different parts of the world.

There has been extensive research on career decision making among medial students, including those from LMICs in Africa and Asia. The existing studies mainly focus on the decisions they have made or plan to make, particularly the decisions of specialty choice and practice location choice (Azu et al., 2013; Chuenkongkaew et al., 2016; Deressa & Azazh, 2012; Sawaf et al., 2018), as well as the factors influencing these different career decisions (Anand & Sankaran, 2019; Eze et al., 2011; Kotha et al., 2012; Sapkota & Amatya, 2015; Syed et al., 2008). On the other hand, despite the proliferation of IMSs on a global basis, only a limited number of studies available have looked at IMSs’ career decision making, which also mainly reports on their decisions on specialty choices and practice location choices (Chen et al., 2019; Hawthorne & Hamilton, 2010; Li & Sun, 2019; Li et al., 2023; McGrail et al., 2019; Mospan & Slipchuk, 2020).

What career choices people make (the result) and how they make career choices (the process) are different (Tang, 2019). As demonstrated in the previous literature, both domestic medical students (Anand & Sankaran, 2019; Mwachaka & Mbugua, 2010) and IMSs (Li et al., 2023) have shown varying degrees of undecidedness towards their career choices, which indicates the apparent career decision-making difficulties these students may experience are not inconsequential and warrant further investigation. Identification of the sources for career undecidedness can help medical students formulate strategies to overcome the problems and provide insights for institutions to develop targeted career guidance service (Lee et al., 2022), so as to enable the students to make more informed career decisions.

Compared to medical graduates trained domestically, international medical programme graduates have been reported to face additional challenges related to cross-border transitions around residency, licensing, employment, and healthcare system accustomedness, regardless of returning or staying abroad (Anjali et al., 2016; Brouwer et al., 2022; Chur-Hansen, 2004; Patel & Araya, 1992). Therefore, the career guidance for domestic medical students may not well apply for IMSs’ contexts. It is then imperative to understand IMSs’ career decision-making process in depth, so that career-related supports and interventions that are oriented to IMSs’ needs can be designed and provided. Given this, the aims of the current study were to explore how China-educated IMSs made career decisions, and what challenges and coping strategies were involved during their career decision-making process.

### Theoretical framework

The Cognitive Information Processing (CIP) theory is a well-established approach to career decision making and career problem solving (Sampson et al., 2020). Unlike most career decision-making theories which primarily describe the career decision-making process, CIP theory focuses on the acquisition of career problem-solving and decision-making competence, so as to help individuals become better and independent career decision makers (Arthur & McMahon, 2019). As in the current study we not only aimed to interpret IMSs’ career decision-making process, but also identify the career decision-making challenges and propound coping strategies in response to the challenges, we considered CIP theory most suited to our research aims. The CIP approach has been effectively incorporated into designing career interventions among various student groups in different countries (Hirschi & Läge, 2008; Osborn et al., 2016; Qamaria & Astuti, 2021; Reardon & Wright, 1999), which provides evidence for its potential in interpreting and facilitating the career decision-making process of IMSs.

**Fig. 1:**
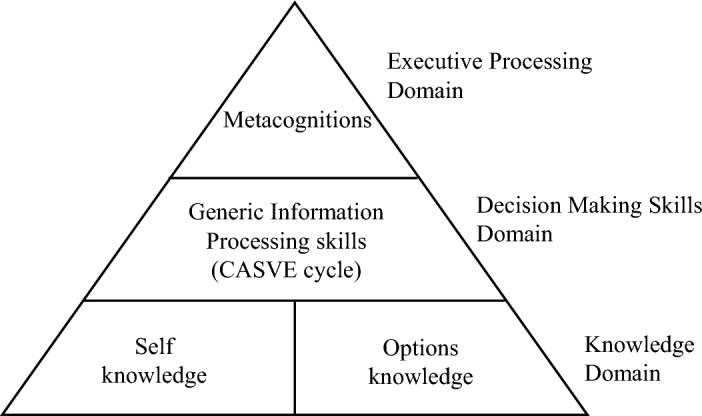
The Pyramid of Information Processing Theory domains

The foundational elements of the CIP theory contain the Pyramid of Information Processing and the CASVE cycle (Arthur & McMahon, 2019). The Pyramid of Information Processing concerns the contents involved in career decision making, which are layered into three domains: knowledge domain, decision-making skills domain, and metacognitions domain, which is presented in Fig. 1 adapted from Sampson et al. (2020). At the base level of the pyramid, there is the knowledge domain, including self-knowledge (individual’s values, interests, abilities and employment preferences) and options knowledge (knowledge of career options and a schema to organise this knowledge) (Sampson et al., 2020). The second level of the pyramid is the decision-making skills domain, which contains the decision-making process as well as the factors that influence the decision-making process (Arthur & McMahon, 2019). At the apex of the pyramid, there is the executive processing domain, which involves metacognitions (thinking about one’s own thinking patterns) in terms of self-talk, self-awareness, and control (Hirschi & Läge, 2008; Reardon & Wright, 1999).

The CASVE cycle is placed in the middle level of the pyramid structure, that is, the decision-making domain, which presents a detailed model for the decision-making process (Arthur & McMahon, 2019). The CASVE cycle comprises five steps: starting from Communication (realising the gap between the present career situation and the desired career situation), followed by Analysis (understanding themselves and their options), Synthesis (elaborating to expand the options and crystalising to narrow the options), Valuing (selecting a first career choice), Execution (testing the preferred option in reality), and back to Communication (revisiting the original gap and evaluating the choice made) (Arthur & McMahon, 2019; Sampson et al., 2020). According to the CIP theory, the career decision-making process can be facilitated if people have a higher level of decision-making capabilities to positively engage in undertaking the task of career problem solving (Sampson et al., 2020). On the other hand, the career decision-making process can be impeded if people have a higher level of complexity concerns, which may relate to contextual issues including family, social, economic and organisational factors (Sampson et al., 2020).

By reviewing the studies on medical students’ career decision making challenges or difficulties, we were able to identify some commonly cited problems in the existing literature, such as a lack of relevant information, generalised indecisiveness, external conflicts, and dysfunctional thoughts (Alibhai et al., 2023; An & Lee, 2017; Chen et al., 2021; Lee et al., 2022; Richard et al., 2007; Zhu et al., 2021). The convergence of these career decision-making challenges in previous literature and the key components in the CIP theory indicates the suitability of applying the CIP theory to study the career decision-making process and decision-making problems among the population of medical students. Additionally, the CIP theory can also serve as a promising framework to shed lights on the design of a structured career programme helping IMSs to solve the career problems.

## Research questions

We conducted this study to address the following research questions:

RQ1. What is the process of career decision making among China-educated IMSs?

RQ2. What particular challenges do they encounter during their career decision-making process?

RQ3. What strategies do they propose in response to the challenges?

## Methods

### Study design

This was an exploratory qualitative study with data gathered using one-on-one interviews with senior-year IMSs educated in China. We drew on the CIP theory (Sampson et al., 2004, 2020) to guide our study to explore IMSs’ career decision making process, as well as the challenges they encountered and the strategies they used during the career decision-making process.

### Setting

The study was conducted at an independent regional medical university in east China. This university accommodates international students from nearly 50 countries mainly in Africa and Asia and therefore enables a diverse sample in terms of students’ countries of origin. Each year, approximately 60 IMSs commence their studies in an English-taught undergraduate medical programme in this university. The programme is 6 years long. The first to fifth years are lecture-based coursework with lab teaching and bedside training, and the sixth year is the clinical rotation internship, which IMSs can choose to do either in China or in other countries with approval. IMSs will be able to obtain the degree of clinical medicine upon successful completion of their academic studies and the clinical rotation internship.

### Participants

Participants were selected from those in their fifth and sixth years of study, as they had adequate experience in medical education as well as clinical training and tended to consider career prospects more seriously (Kuteesa et al., 2021). To develop as many perspectives as possible (Creswell, 2012), we created a list for potential participants using the purposeful sampling strategy, with the help of staff at the university to maximise the diversity of the sample, mainly according to participants’ gender, year of study, country of origin, and current residence (some IMSs were residing outside China because they were doing internship in another country or they were receiving online education due to travel restrictions then). The participants were invited according to the list via WeChat, a social networking application which is widely used among domestic as well as international students in China. An interview appointment was scheduled as soon as the potential participant expressed willingness to participate.

Altogether, 38 IMSs were approached, among whom 14 did not respond and 4 refused our invitation due to being too busy or being sick at the time. A total of 20 IMSs were interviewed. Gender, nationality and study years of the participants were well balanced (Table 1). The number of IMSs of each nationality was large enough to allow their identity not to be revealed. In our sample, Asian students were mainly from India, while African students were dispersed in various countries. We considered this sample adequate as we believed the themes developed from this sample could provide sufficient information for answering our research questions (Varpio et al., 2017).

**Table 1:** Characteristics of IMS participants

Characteristics	Fifth year	Sixth year	Total
*Gender*			
Female	5	5	10
Male	4	6	10
*Home country*			
Asian countries			10
Bangladesh	1	1	
India	3	4	
Yemen		1	
African countries			10
Botswana		1	
Kenya		1	
Morocco	1		
Nigeria	1	1	
Uganda		1	
Zambia	1	1	
Zimbabwe	2		
*Current residence*			
Home country	5	7	12
China	4	3	7
Egypt (for internship)		1	1

### Data collection

One researcher (WL) conducted semi-structured interviews with all participants from July to August 2022. As the participants were residing in different countries, the interviews were conducted virtually via Zoom. A Zoom link and informed consent form were sent to participants via email. In addition, an information sheet with a short summary of the research project was also provided to inform them of why this topic was important to discuss and what would be discussed during the interview, which could help build rapport with the participants (McGrath et al., 2019). Participants were allowed to choose whether the interview would be audio/video recorded or written notes taken. Interviews were conducted in English and lasted approximately 40–60 min. During the interviews, field notes were kept and live captions in Zoom were enabled upon permission from the participants to aid better communication. The interview guide included open-ended questions about the participants’ future career plan, experience of making career decisions, challenges during career decision-making process, strategies used for coping with the challenges, supports needed, as well as some specific probes on certainty towards career plan, current stage of career decision making, and challenges and strategies related to CIP pyramid domains (Hsieh & Shannon, 2005), which was informed by the pre-conducted literature reviews and our theoretical framework (Supplementary Material 1). These were first piloted with 2 IMSs to assess whether the questions were adequate and well understood.

Among 20 participants, 15 participants preferred their interviews to be audio/video recorded, whose recordings were transcribed verbatim into text, with reference to the transcripts produced by Zoom. Five participants preferred written notes to be taken, so the field notes as well as the text files of the live captions produced by Zoom were used for transcribing. In this way, accuracy and rigour of the transcripts were better ensured. All the transcripts were de-identified and coded using NVivo (version 12.6.1 for Windows).

### Data analysis

We used the directed qualitative content analysis for the current study, as we had the CIP theory as our guiding framework and intended to investigate the pertinence of the preexisting framework and the new phenomenon (Hsieh & Shannon, 2005). The five stages of career decision making in the CIP CASVE cycle served as our initial framework to identify the career decision-making stages of IMSs (RQ1). In addition, the three fundamental concepts of knowledge, decision-making skills, and metacognition in the CIP pyramid structure served as the key themes of initial coding categories for career decision-making challenges (RQ2) and coping strategies (RQ3).

One researcher (WL) coded the data following the coding strategy proposed by Hsieh and Shannon (2005): (1) reading the transcripts, (2) highlighting all the texts considered relevant to the research questions, and (3) categorising or subcategorising the marked texts using the initial coding scheme or creating new codes. Coding was conducted three times for each transcript, with a different focus each time in regard to the three research questions. The trustworthiness of the coding process was facilitated by discussions within the research team on a regular basis. Codes were added, refined, or removed, subject to the agreement by all the team members and finalised upon the team consensus. To improve accuracy and credibility of our data interpretation, a summary of our findings was sent to the participants via the method of member checking (Creswell, 2012), and their feedback did not lead to any changes.

### Methodological considerations

Our research team consisted of members from different disciplines, all of whom had rich experiences in international education or medical education. WL and HS have been intensively involved in administration and teaching in international medical programmes in China’s context. AK has been doing research and teaching in health sciences. RG is an education researcher with special interest in career counselling, who has been teaching and doing research related to international students. As such, the interviewer could incorporate the knowledge and competencies in the field to better comprehend the circumstances of the interviewees (McGrath et al., 2019). Moreover, the team members complementary expertise benefited the study by virtue of the diverse backgrounds, multiple perspectives and rich experience. As two researchers (WL and HS) had been engaged with IMSs in China for a number of years, our previous backgrounds and experiences not only enabled familiarity and inspiration on the research topic, but also shaped the assumptions related to the research (Brouwer et al., 2022). Therefore, we valued the opinions and continuous input from AK and RG, who contributed to balancing the perspectives when there were divergent interpretations of data and stimulating further explorations when findings were different from the initial assumptions (Varpio et al., 2017).

English has been chosen as the language for conducting the interviews. To be admitted into China’s institutions for international medical programmes, candidates are commonly required to either originate from English speaking countries, or meet certain English language standards. Therefore, our participants are expected to speak fluent English. In the meantime, the interviewer is bilingual and can speak fluent English and Chinese. Nevertheless, as the participants and the interviewer are from different countries, they may encounter language barriers due to issues such as cultural differences or accents. To reduce the potential influence from language barriers, the interviewer would check with the participants during the interview if they found any questions which were unclear. The interviewer would also use some techniques such as paraphrasing what she heard from the participants and confirming with them the meanings they intended to express.

### Ethical considerations

This study was reviewed and approved by the ethics review committee at The University of Queensland, Australia (2022/HE001071). An approval letter for interviews was obtained from the university where the participants were enrolled. All the participants gave informed consent. Member checks were employed for obtaining participants’ approval to use direct quotations and guaranteeing anonymity (Thomas, 2017).

## Results

During the interviews, while describing their experiences, participants tended to be aware of their role as an IMS educated in China. However, they seemed to regard this IMS role as introducing complications or creating additional challenges for their career decision making, with some participants expressing it in an overt way while others were more covert. To facilitate clarity and readability, we presented our findings according to our research questions, and emphasised the information that was particularly relevant to IMSs.

### Career decision-making process of IMSs

The career decision-making process of our participants, despite their genders and nationalities, seemed to generally follow the CASVE cycle in CIP theory. When they were aware of the need to make a career choice, they tended to revisit the idea of what to do and where to go for their future career (C, Communication). Following that, they started to explore themselves from various perspectives, and the possible options within and outside the medical profession as well as options to return home or migrate to a foreign country (A, Analysis). They then developed some further options and rejected some, leaving themselves with a list of alternatives (S, Synthesis), from which they chose the best one (V, Valuing). Following this, they would actually take some actions to test, prepare for, or seek internship in order to implement their choice (E, Execution). Some of the quotations from the participants representing the different stages during the career decision-making process were presented below:

- (Communication) What other jobs can I get with an MBBS degree that’s not being a doctor? (Participant 01)
- (Communication) So, when normally I had planned, at this stage, I would go visit the countries of my choice and make plans on how I was going to move there. (Participant 17)
- (Analysis-self) So far, I’m just interested in internal medicine as a general doctor. But through time and probably I might find that specialisation that I get interested in. (Participant 02)
- (Analysis-options) I’m still growing. I still have other options. It doesn’t mean I’m a doctor, it doesn’t mean it’s the only thing I can do. I have other things I can explore as well. I can do businesses. I can, like being said, open hospitals, then help more people, I also want to open orphanages. It helps a lot of orphans and other people in it...I believe I can also do other things, not only medicine. (Participant 15)
- (Synthesis) It simply grows. As you grow up you pick, and you kill some. You grow some, your kill some. You grow some, you kill some. By the end of the day, you have this small criterion to pick from, and you have like three alternatives, so you just pick and try. (Participant 05)
- (Valuing) The first being that they both are English speaking countries…I have grown up speaking English and I’m comfortable with it, and the second aspect is I’ve got family in both of the countries, like extended family… so probably settling in wouldn’t be as hard, because I would have a community to talk to, and to share my life with, once I get there, rather than going to a place where I might not know anyone there, like totally starting over, and I might experience very concerning degree of culture shock, while also trying to like manage my work life, and all of that. (Participant 11)
- (Execution) Now I’m doing an internship at a referral hospital in my country. I’m working at this referral hospital. My plan is to continue working at the referral hospital in my country after I do graduate from China. (Participant 07)

However, it was noted that IMSs’ decision making usually involved additional rounds of the CASVE cycle. As mobile students with visa limitations, they were faced with urgent migration decisions to make regarding internship or immediate post-graduation locations, which their domestic medical counterparts probably did not find so pressing. Therefore, they tended to spilt their career plans into short-term and long-terms ones, and focused on the more urgent choices before going through the cycle again for further choices in regard of migration as well as specialisation:

- I think it’s better if you go and do your internship and go be registered as a doctor where you come from, and then venture out into for intention again with education. (Participant 07)
- With my six-year degree program, there is a final year, sixth year which is an internship year, so I’ve just been looking at a few countries that I can go to, which probably would decide if I then want to stay there for, like after I graduate, of course. (Participant 11)
- When I start my master’s in internal medicine, we will be learning all the things. At that time, I will think like what to do, for this subspecialty, go to nephrology, or neurology, or cardiology…So, like once I do my internal medicine for three years, then I will know which to take further, subspeciality, and then I will do some subspecialty like that. (Participant 06)

Additionally, IMSs tended to consider specialty and migration choices side by side and aimed to achieve an ideal balance between these choices:

- If I end up going to a country like the US or Canada, maybe I would end up doing, more of a general surgery kind of thing, by even ending up in certain surgical field at all, it would be general surgery. But back in my home country with no restrictions, I would like the orthopaedics field, orthopaedic surgery. (Participant 17)

**Fig. 2:**
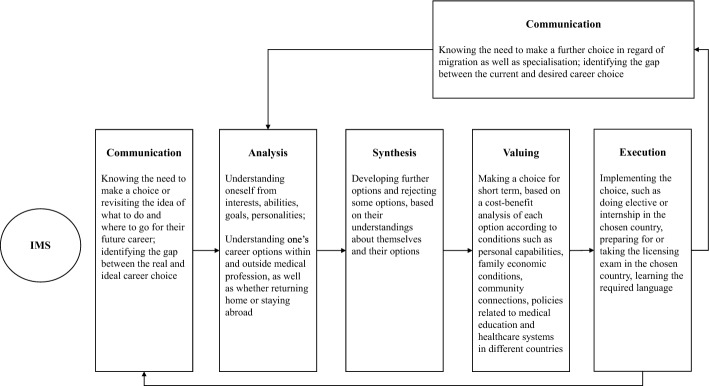
Application of the CASVE cycle for IMSs

Based on our data analysis, we used a figure to map IMSs’ general career decision-making process, which was adapted from the CASVE cycle in the CIP theory (Fig. 2) (Sampson et al., 2020).

### Challenges experienced by IMSs during the process

According to the participants’ descriptions, we noticed they were at different stages of the career decision-making process, and there were challenges which blocked them at a certain point which prevented them from entering the next stage or reaching the career decision. Based on the CIP framework as well as the interview data, we identified the key challenges, which were categorised into the CIP pyramid structures. To facilitate specificity of our findings, we presented more detailed information specific to IMSs in this section and listed the complete entries of all the challenges in Table 2.

**Table 2:** Challenges for IMSs’ career decision making and corresponding strategies

CIP pyramid levels	Challenges	Strategies applied or recommended	
		Personal aspect	Institutional aspect
Base level: knowledge domain	*Lacking knowledge about oneself* • Needing to know more about one’s interests • Needing to know more about one’s capability • Needing to know more about one’s goal • Unsure about one’s suitability for the desired career	• Seeking advice from people who know the decisionmaker well	
	*Lacking knowledge about career options* • Hard to get information about career • Hard to get information about recognition of overseas medical degree • Needing more clinical experience • Needing more resources of career guidance	• Obtaining the needed information using various ways • Accumulating practical experiences	• Providing knowledge related to career in various forms • Providing resources about clinical practice • Creating communications with peers and faculties • Fostering mentorship from professionals • Providing continuous help to alumni
Middle level: career decision-making skills domain	*Lacking career decision-making skills* • Being indecisive towards the desired career • Being indecisive among the career options • Being indecisive in personality	• Thinking logically and sorting out the difficulties • Standing firm for one’s original aspiration • Eliminating the unlikely options • Confirming the indecisiveness	• Teaching effective career decision making skills
	*Facing contextual complexities that limit the career decision-making process* • Facing extra procedures or disadvantages related to overseas medical education • Facing extra uncertainties and problems created by the COVID-19 pandemic • Having financial concerns for the desired career • Unsupported by family	• Managing the finance • Improving one’s competence • Communicating with family to reach a consensus	• Providing help with issues related to the COVID-19 pandemic • Providing the needed support related to overseas medical education • Making adjustments to curriculum and extra-curriculum activities
Top level: metacognition domain	*Feeling unwilling to begin the process of career decision making* • Overwhelmed with study burden • Overwhelmed with internship duties • Psychologically unready to do so	• Increasing self-motivation • Reminding oneself about the inevitable struggles in advance • Setting a target to push oneself forward	• Raising students’ awareness regarding career decision making
	*Being negative about career decision making* • Doubting one’s competence in achieving the desired career goals • Thinking about obstacles a lot • Feeling anxious about making a career choice • Denying the choice made by oneself	• Thinking positively and being unafraid to make mistakes • Seeking comfort from outside • Leaving it for now and revisiting later • Doing physical exercises to relax • Having a flexible timeframe • Having a backup plan	• Providing service related to mental health

#### Knowledge domain: base level in CIP pyramid

IMSs expressed a need for knowing more about themselves, including their interests, capabilities, goals, and suitability for the desired career. They particularly emphasised the importance of understanding their capability to apply what they had learned in China to different settings:

- My plan is to go back home, stay there for a few years, and then if I’m capable of coming back and doing, I can get specialised. Yeah. Yeah, but I want to implement what I’ve studied so far, so to see how it goes there. (Participant 02)

As mobile students, they tended to consider their migration plans after graduation, which made them realise the need to figure out their higher priority among their career or life goals, such as getting married versus staying abroad for career ambition/returning home to work as a female doctor:

- I do think about it, like okay, if I end up in a foreign country, I might not then, you know, meet someone from home, you never know. (Participant 11)
- Men would be intimidated by you, nobody wants to talk to you, men they won’t want to marry you, because they’ll be intimidated, or they’ll be scared. A lot of people think doctors are smart, so they will be scared by that. (Participant 15)

Besides the challenges in the self-knowledge area, participants also described challenges in the area of knowledge about career options, including the career-related information and the ways of obtaining the information. Studying in China, they needed to collect information about further training opportunities and career pathways in their destination countries, which created additional inconveniences for them as they were not physically there:

- So it’s definitely hard, because information is hard to come by, if you’re just always looking online. Sometimes, searching online is not as clear as you think it is. (Participant 11)

Furthermore, as foreign-trained medical students, they had extra difficulties in getting information about recognition of overseas medical degrees in their home countries or other destination countries, because they were not the mainstream medical graduates in those countries, and related information seemed not adequately provided:

- So they asked me to go to the capital before like getting the information and even though, when you go there, they don’t give you like enough information. They be like we don’t know much about degrees from China, because not a lot of students from my country go to China, especially for medicine. Like they go for other like fields like economy and stuff but not for medicine, so they don’t have enough information and that’s like a struggle for us. (Participant 03)

#### Decision-making skills domain: middle level in CIP pyramid

Some of challenges our participants articulated echoed to the middle domain in CIP pyramid, which related to lack of skills that were needed for career decision making and the contextual complexities that impeded the career decision-making process.

Our participants deemed lack of the needed career decision-making skills limited their career decision-making process, exemplified by the indecisiveness of ascertaining the desired career or choosing one from the options. For those who had already chosen the most desired career, from time to time they were of two minds about whether they really wanted this career, or they could not resist the idea that their minds would change over time. For those who were still trying to choose, they might experience “confusion of selecting various careers”, because there were too many options there or too many factors to consider. They might feel it was too difficult to choose only one option because they were “interested in multiple things”, or they did not know what to do because they had an indecisive “personality”.

- I’m also interested in many things. So, choosing to specialise in only one thing is difficult. I do find both of them interesting, so it’s also very difficult to choose just one. (Participant 09)

Apart from the lack of decision-making skills, our participants also expressed a diversity of contextual complexities that limited their career decision-making process, which related to family (disagreement with family members), society (potential bias from employers), economic (financial concerns), and organisational factors (the COVID-19 pandemic, policies in medical education and healthcare systems in different countries).

Some of the participants were concerned about the possible “stigma” that might cause potential bias from the employers due to the “the location of the school”:

- There is an unwillingness to hire doctors that schooled in what they view as developing countries. (Participant 17)

Some participants regarded the extra procedures related to the accreditation of their overseas medical education as one of the contextual complexities, such as doing an additional internship and writing licensing exams oriented to foreign-trained medical graduates:

- After the internship I still need to do the board exams a year before I now do another internship. That’s just the policy that they have, you have to do board exams first before you do another internship. So, for now, it’s considered an elective internship. (Participant 09)
- The licensing exam in my home country is not an easier thing. It is much harder. I think the past percentage in this licensing exam is very less. I know many of my seniors, they didn’t yet pass this licensing exam. That too, seeing many of my seniors who were excellent, excelled in studies didn’t pass this licensing exam. Those who passed this licensing exam, the first attempt is very rare. So, I’m afraid of that and that makes all of my career decision in a hard way. (Participant 18)

Another concern raised by some participants was that they seemed to be in a less favourable position compared to the domestically-trained medical students, from aspects of theoretical knowledge as well as clinical skills related to different medical education systems, due to distinctions in disease spectrum or devices used for clinical training, which was a matter of difference in teaching rather than the quality of teaching:

- Those are some of the challenges that I face, I think, saying, okay, so what kind of diseases are more endemic in these areas and what kind of information, because medicine is a lot of stuff, you can’t know everything definitely, but the thing is that you definitely need to know that other students in those countries are being taught. (Participant 11)
- Number one point to be that different countries train their doctors differently. So, this style of teaching and the syllabus might be the same, but the ways they go about it are different. So, for example, underdeveloped countries in Africa don’t train the doctors to rely on medical equipment as much as developed countries would. (Participant 17)

The COVID-19 pandemic undoubtedly caused extra uncertainties and problems for their career decision making as IMSs. Due to the global travel ban as well as occasional suspension of clinical trainings in China, IMSs had to receive prolonged online education and missed many offline practical classes. This not only reduced their understandings of different specialties and confidence in clinical competence, but also forced them to reconsider their migration destinations, as some countries made it clear that they did not accept “online medical students”. For those who were interested in staying in China, the travel restrictions deprived them of the opportunities to immerse themselves in China’s environment, know the policies and study the Chinese language; for those who aimed to migrate to other foreign countries, they were frustrated with the inability to complete the potential elective or internship in their destination countries. These created extra challenges for them to make decisions.

- I had planned before COVID to go on an internship programme, working around Europe. But I can’t go anywhere because of COVID. (Participant 17)

#### Metacognition domain: top level in CIP pyramid

Some of the challenges participants encountered belonged to the executive processing domain located at the apex of the pyramid, which negatively influenced the commencement or progress of whole career decision-making process. This suggested the possible psychological maladjustment risks among IMSs.

Some participants indicated an unwillingness to start the career decision making. This was due to a general psychological reluctance or their concerns with extra academic burden of studying abroad or internship accommodation issues back home:

- For me, yes, making decisions about the future is very hard for me, mainly because I’m a person who lives in the present moment. So it’s very difficult for me to think far into the future. (Participant 01)
- You have to look at the fact that you have to put two times the efforts into schooling, because you have to study syllabus for two different countries. (Participant 17)
- And I’ve noticed that our supervisors and our attendings in the hospital, they leave a lot more practical work to us, they make more of the decisions and we help with the ward work. (Participant 07)

At this executive level, some participants also added challenges related to their negative thoughts. They doubted their ability in achieving the desired career goals, thought about obstacles a lot, felt anxious about making a career choice, or denied the choice made by themselves:

- But like the problem is the negative force that someone would have. The thoughts that you have, the mindset that you have is negative. You’re thinking about obstacles. (Participant 02)
- That also hinders, making a decision difficult for me. That anxiety. So, it’s more like a mental state, I guess, you could say that. (Participant 14)
- But sometimes I feel like, is it a wrong decision? (Participant 04)

### Strategies implemented or recommended by IMSs in response to the challenges

The participants had successfully “beaten” many of the challenges for career decision making and arrived where they were. In the interviews, they shared the effective strategies which they applied during the process. They also voiced the need for help with some unsolved issues, for which they proposed some interventions they hoped to receive from the institution. To clarify the strategies that needed to be conducted at different levels, we listed the strategies from personal and institution aspects in Table 2, in response to the challenges we identified earlier. Quotations describing strategies proposed by IMSs were available in Supplementary Material 2.

## Discussion

We conducted a qualitative study to canvass the career decision-making process of African and Asian IMSs educated in China under the guiding framework of the CIP theory. We found that generally IMSs followed the career decision-making cycle steps in CIP theory; however, the urgency of migration decisions, coupled with considerations of medical specialties, appeared to add complications to their career trajectories. During their career decision-making process, they encountered general challenges as well as challenges specific to the population of IMSs, so they seemed to have, as cited from one of our participants, extra hoops to jump through, extra plans to do, extra steps to take. Notably, our findings verify and extend the CIP framework in the context of China-educated African and Asian IMSs by elucidating their unique experiences and intricacies inherent in their career decision making. By categorising the career decision-making challenges and coping strategies under the guidance of the CIP framework, our study contributes a nuanced understanding of how our particular cultural and educational contexts shape and modify the applicability of the CIP theory, thereby enriching the theoretical landscape in this aspect.

Our study suggested the importance of migration choice IMSs placed in their career plans. This finding is not surprising, as physicians from LMICs in Africa and Asia have a high emigration intention to HICs (Syed et al., 2008), and IMSs’ temporary resident permits might also remind them of the urgency in the migration choice. Having specialty and migration to consider simultaneously can elevate the complications of IMSs’ career decision making, as these two aspects are not paralleled but interrelated. Preferring a competitive specialty may lead them to a concession in practice locations, while prioritising migration to HICs may force them to lower the specialty expectations by choosing a less attractive field (Li et al., 2023). This was also reflected in our findings. However, the existing literature related to career guidance for medical students demonstrates an overwhelming focus on the specialty choice (Ock et al., 2020; Richard et al., 2007), leaving the guidance on migration choice a neglected aspect. Our study thus signifies a need to incorporate migration decision making with specialty decision making when designing the career interventions for IMSs in China.

Some of the career decision-making challenges encountered by our IMS participants are also well documented in previous research conducted among domestic medical students, namely lack of career knowledge, lack of self-knowledge, indecisiveness, external conflicts/barriers, lack of readiness, and distressful thoughts. These challenges have been investigated wholly or partially in studies from the US (Richard et al., 2007), Malaysia (Chen et al., 2021), Korea (An & Lee, 2017; Lee et al., 2022), Canada (Alibhai et al., 2023) and China (Zhu et al., 2021). However, these documented challenges are rather general ones, and the additional difficulties identified by IMSs lead us to further contemplate the difficulties the target population have to face and the career support that can be provided to cater to their specific needs.

As IMSs, a minority group compared to their in-country counterparts, our participants conveyed greater difficulties in obtaining the needed career knowledge, in terms of content (information about overseas medical degree in their home countries can be obscure) as well as the method (online searching can hit dead ends). This finding suggests that similar interviews or surveys could also be conducted in other universities in China to further confirm whether these are common concerns among IMSs. As the issue expressed by our participants regarding the public unavailability of recognition policies of China’s medical degrees in their home countries seems hard to be addressed at the individual level, we thus suggest that the education institutions may shoulder more responsibilities in assisting their IMSs with access to the information as an official authority, such as providing resources to help communicate with the relevant officers in IMSs’ home countries for a more direct and unambiguous answer. In addition, the restrictions in digital infrastructure and website construction in less privileged countries can lead to these countries’ limited capacity of offering resources online (Foster et al., 2021), which serves as a possible reason contributing to our participants’ frustrating experiences of online searching. Therefore, an online platform with some useful career information for IMSs may be provided by the education institutions. In fact, a national league of IMS-recruiting universities in China, China International Medical Education Committee, has already been established, which mainly focuses on the quality of teaching and training of IMSs (Li et al., 2020). This platform therefore provides possibilities for more collaborations among the universities for joint efforts in providing career information for IMSs in China at a national level.

Lack of self-knowledge about capability was highlighted by our participants, mainly referring to the capability in theoretical exams as well as in clinical practice. This is understandable, as although IMSs can well evaluate their theoretical and clinical performance in China’s context according the results of their school exams and feedback from their teachers, they may wonder what if the context changes to their home countries or somewhere else. These concerns are solvable. First, training sessions about the knowledge in foreign medical licensing exams can be organised and mock exams can also be arranged accordingly, so as to strengthen IMSs’ understandings about their competence in theoretical exams outside China. Second, clinical elective opportunities in IMSs’ destination countries can be sought by students themselves or with assistance from their education institutions, as elective experiences can serve as an effective way to explore their capabilities in the corresponding clinical environment (Chur-Hansen, 2004).

Contextual complexities pertaining to an overseas medical degree were perceived to pose challenges for career decision making by our participants. Their considerations are supported from evidence in previous literature (Brouwer et al., 2022). The unfavourable experiences of the overseas trained medical graduates have been widely discussed, in terms of registration, licensure and accreditation, and these barriers may hamper, jeopardise, or even devastate their medical career plans (Motala & Van Wyk, 2019). In fact, some of these barriers are inevitable, as they are related to different medical education and healthcare systems, which inherently disadvantage medical graduates who have received their education abroad. Apart from the structural barriers all IMSs have to face, some obstacles are specific to IMSs’ home countries. For example, the pass rate of the Foreign Medical Graduate Examination screening test, which Indian IMSs need to complete, is as low as 25%, and those who fail in the tests are unable to enter the Indian health system (Anjali et al., 2016). For IMSs from South Africa, their home country has ceased to provide opportunities for overseas trained medical graduates to write board exam during 2020 to 2023, due to government’s lack of accommodation capacity for the examinees (Ntengento, 2023). By comparison, some obstacles are related to IMSs’ education country, China, a LMIC. A study on Malaysian medical students studying in Australia has also reported a concern from the participants about their professional knowledge and clinical competence due to the different training and working styles between the two countries (Chur-Hansen, 2004), but for IMSs studying in LMICs, this concern is not only raised by students themselves, but also by their potential employers, which may negatively influence their competitiveness in the labour market (Anjali et al., 2016). These external factors increase IMSs’ dilemma in weighing up the pros and cons while making career decisions.

Some of our participants expressed negative thoughts towards career decision making, such as low motivation and high anxiety. Compared to those who study in their home countries and thus live closer to their family, our participants can experience more inconvenience in receiving the support from their close ones. Besides, due to the language and cultural barriers, our participants as IMSs may also tend to underutilise the mental health counselling services provided by the education institutions or the local hospitals (Newton et al., 2021). Therefore, it is pivotal to include related counselling services in the career guidance for IMSs, which are recommended to be conducted in English by well-trained professionals.

### A proposal of tiered career guidance intervention programme

**Fig. 3:**
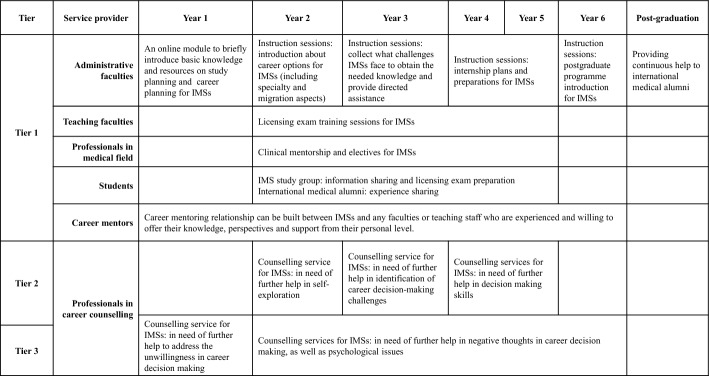
Interventions in different years at medical school

Our study suggests a pressing need for career support for China-educated IMSs from institutional and even national levels. As an initial step to facilitate this endeavour, we tentatively proposed a career guidance intervention programme tailored for China-educated IMSs by referencing to the CIP theory framework, with the identification of their challenges and strategies (Fig. 3). In the proposal, we listed the various interventions which could be implemented in different study years and indicated the potential service providers. We also differentiated three tiers of interventions targeted at different groups of IMSs with varying needs in career decision making, which was informed by the CIP-based differentiated service delivery model (Osborn et al., 2016) and the Multi-Tiered System of Support model (Burns et al., 2016). This differentiation of the career guidance services can maximise the access of the services and optimise the amount and type of the services received by different students (Osborn et al., 2016).

#### Tier 1 intervention: core instructions

Tier 1 interventions are instructions that the whole population of IMSs receive, which corresponds to the base level of knowledge domain in CIP Pyramid Model. In this tier, IMSs can be provided with various sessions to obtain the needed knowledge about career and themselves from various aspects. Core knowledge about licensing exams should also be provided, as its importance has been highlighted by IMSs. In the freshman year, students can be offered an online module about basic knowledge and resources on study and career planning to get preliminary ideas. From the second year through fifth year, information sessions and interactive activities with medical specialists, teaching faculties and classmates can be provided in various forms to help IMSs understand themselves and career options better, develop their interest, and find a career that attract them and suit them most. In the sixth (internship) year, information sessions about postgraduate programmes in China and other countries can be provided to help IMSs make decision regarding further education. Throughout medical school, career mentoring opportunities can be made accessible to all the IMSs, with faculties or staff volunteering to assume the role of a mentor, providing knowledge, offering encouragement, sharing critical perspectives, and raising challenging issues when needed. As suggested by our participants, information sessions regarding career advancement or further training opportunities in China can be provided to alumni to facilitate their career prospects and foster a sense of community. In all the information sessions, both specialty and migration choices should be given due consideration.

#### Tier 2 interventions: additional support

Tier 2 interventions are for the group of IMSs who need further help in general information processing skills, which corresponds to the middle level in the CIP theory pyramid. Our participants experienced different degrees of undecidedness for career decision making, among whom, some were undecided because they lacked sufficient knowledge, which could be rectified with enough information in Tier 1, while some were undecided because they lacked skills in identifying their interests, capabilities or goals, who needed further assistance from professional help. Meanwhile, some others may need additional help to deal with their escalated external complexities. Therefore, we suggest that from second year to fifth year, counselling service can be provided for those who still feel indecisive when they have sufficient information. This service can be provided through small groups, where students with similar challenges in career decision-making skills can participant together.

#### Tier 3 interventions: individualised support

Tier 3 interventions are for the group of IMSs who express negative thoughts towards career decision making, such as overwhelming, fear, and anxiety, or potential mental health issues. This corresponds to the top level in the CIP theory pyramid, which overlooks the whole career decision making process. The targeted group will need remedial approaches to alleviate their difficulties. In year one, if students still feel nervous or unwilling to start the career decision-making process after obtaining the basic information, they can refer to counselling services to seek help. Since the second year, the counselling services can be made available for those who need further help to deal with negative thoughts and related psychological issues in career decision making. Due to the intensive difficulties encountered by the targeted student group as well as the highly private issues that may involve, the services in Tier 3 should be provided individually to cater for customised needs.

## Limitations

Our study has limitations. The relatively small sample size of our study from a single institution limits the individual and institutional diversity, although a strong variability from our sample has been observed based on our findings. Additional data collection from more participants originating from relevant countries in other education institutions may reveal more perspectives to understand the studied phenomenon. As the interviewer and participants are from different cultural backgrounds and they may not be native English speakers, there may exist language and cultural barriers which limit the depth or affect the accuracy of the interpretation. There were five participants who only wanted notes to be taken during the interview, which limits the possibility of verbatim transcription of their texts, although we have adopted techniques such as keeping field notes and enabling the live captions in zoom to minimise this influence.

## Conclusion

Our qualitative study uses the CIP theory to delve into the intricate career decision-making process of IMSs educated in China, substantiating and expanding the application of the CIP theory within the sphere of this particular cultural and educational context. While IMSs generally follow the CIP-based decision-making cycle steps, their unique circumstances lead them through possibly multiple rounds of the cycle. Their specific challenges in career information access, self-capability evaluation, degree accreditation, employment competitiveness and mental states necessitate targeted support for this minority group of medical students. Our study underscores the underestimated significance of integrating migration decision-making into career guidance interventions for IMSs, and contributes to the literature by proposing an evidence-based tiered career guidance intervention programme for IMSs.

## Supplementary Information

Below is the link to the electronic supplementary material.

Supplementary file1 (DOCX 20 KB)

Supplementary file2 (DOCX 28 KB)
