# Supplementary material for: A qualitative study of career decision making among African and Asian international medical students in China: process, challenges, and strategies
Source: Adv Health Sci Educ Theory Pract. 2024 Apr 9;29(5):1711–34. doi: 10.1007/s10459-024-10329-z (PMC11549110; doi:10.1007/s10459-024-10329-z)
Supplement: Supplementary file 1 — Supplementary file1 (DOCX 20 KB) [file 10459_2024_10329_MOESM1_ESM.docx]

**Interview guideline questions**

**Career decision-making process**

1. Can you please share with me some of your thoughts on your future career plan?

- Probe—How would you describe your certainty towards your career plan?

1. Can you please describe some of your experience about how you make your career decisions?

- Probe—What stage would you describe you are currently at regarding career decision making?

**Career decision-making challenges**

1. Do you think making career decisions is easy or hard for you?

- Probe—If easy: Can you share with me why it is easy for you?
- Probe—If hard: Can you share with me why it is hard for you?

1. Have you experienced any challenges during your career decision-making process?

- Probe—If the participant mentions anything related to the Knowledge Domain in CIP theory: Can you tell me more about what kind of knowledge that you lack and how it poses challenges to your career decision making?
- Probe—If the participant mentions anything related to the Decision-making Domain in CIP theory: What skills do you think you lack for making decisions? What barriers have you encountered which prevent you from proceeding with your decision-making process?
- Probe—If the participant mentions anything related to the Metacognitions Domain in CIP theory: Can you tell me more about why you have such thoughts and how those may affect your career decision making?

1. Are there any other challenges that you can think of?

**Strategies to cope with the career decision-making challenges**

1. What are the strategies that you have employed to cope with the challenges that you have encountered?

- Probe—If the participants have mentioned any challenges in the Knowledge Domain previously: What methods have you tried to obtain the knowledge or information that you lack?
- Probe—If the participants have mentioned any challenges in the Decision-making Domain previously: What methods have you tried if you feel you lack skills in dealing with challenges in decision making?
- Probe—If the participants have mentioned any challenges in the Metacognitions Domain previously: What would you do if you find you have such thoughts?

1. Are there any challenges that are still unsolved and you may need help or support from the outside sources?
2. Are there any other help or supports you may need to receive from your education institution?
