# Supplementary material for: A qualitative study of career decision making among African and Asian international medical students in China: process, challenges, and strategies
Source: Adv Health Sci Educ Theory Pract. 2024 Apr 9;29(5):1711–34. doi: 10.1007/s10459-024-10329-z (PMC11549110; doi:10.1007/s10459-024-10329-z)
Supplement: Supplementary file 2 — Supplementary file2 (DOCX 28 KB) [file 10459_2024_10329_MOESM2_ESM.docx]

**Supplementary Document** Quotations describing strategies proposed by IMSs

| **Strategies** | **Illustrative quotations** |
| --- | --- |
| Seeking advice from people who know the decisionmaker well | - I think I’ll ask friends for advice or suggestions, or my family. Though they wouldn’t understand that much. But I will try my best to ask them and take their opinion. (Participant 20) - It’s like getting advice from your parents. Like the parents are your first teachers. So, getting the best advice is from them. (Participant 08) |
| Obtaining the needed information using various ways | - Since I wanted to do either the master’s, what I usually do is I e-mail certain universities and ask them if they take would take me as MBBS students and that usually works. (Participant 01) - By talking to professionals in the specific speciality that I’m interested in. (Participant 09) |
| Accumulating practical experiences | - That’s why I’m not confirming. I need to complete my internship period and work a little bit, so that I can acquire the needed information, the needed knowledge to compete in this field. (Participant 18) - I think for a start I need to be patient and gain as much perspective as I can in every specialty, so that I can gauge them with knowledge, with as much knowledge as I can. So, patients, I have to practice patients in that, and get as much perspective as I can, so that when I do finally sit down to make a decision. (Participant 16) |
| Providing knowledge related to career in various forms | - I feel like the school should give more insights about all those other specialty programmes, like specialty programmes that are in demand and not too competitive to get into. That’s what I feel. So, providing the information of other specialties that may be a new, let’s say, I heard recently from a doctor, like a lot of people like now and are now trying to get into interventional radiology, a lot of people, because it’s like, you know, it’s quiet captivating to the people in the medical field, interventional radiology, you know there’s so many new imaging procedures, techniques and things like that. (Participant 14) - So that I would appreciate if we could get from the school and also to like information, like knowing what else can you do with an MBBS degree or with the nursing degree after you graduate other than being a nurse or being a doctor, information like that. Or what else can you do after you have the degree within medicine itself? What else can you do whether it's research, being a professor, and how to do that as well, That type of thing. (Participant 01) |
| Providing resources about clinical practice | - I wish, if, like our school has like you know, an arrangement with other countries to do the internship. (Participant 03) - Also maybe ward rounds for particular students, it can be in cardiac department, then the students who are interested in ward rounds can participate in that particular department, the next can be something like just what we do, normally, but now the whole day will be focused on one particular department and students from any other year can join the rounds, so that people can make their decisions at early year. (Participant 09) |
| Creating communications with peers and faculties | - Then the other thing would be helping students to have study groups. And study groups that would actually help them and then at the end of the month, the students come up with an idea, even though it may be helpful to this call or not, as long as it's medically oriented, for example, they have trade what they have learned in this licensing, knowing this research publications, and put it together. (Participant 05) - It would be really great for, if the university could take like a certain approach where they integrate the students and ask them like what do they need, what kind of help maybe they need from seniors, graduates, they have like a meeting with the university they tell them. Okay, so in this year we felt like we need this and this, next year we need this, and these are some of the things that then can be implemented for the coming years. (Participant 11) |
| Fostering mentorship from professionals | - I think mentorship would be a very good idea. For I know a lot of students in school, who want to be cardiologists, but it's very hard to find a cardiologist who is willing to mentor them. I think they should be like mentors in each department, so that students who are interested could come up to them and ask them questions in that particular field. (Participant 09) - And you know, or invite prospective countries that people want to go, to invite the various professors there, or people that have undergone those processes of moving from being an international student to a practitioner in this country, and basically explain to us, okay, this is how you do it, guys, there's a certain exam like this, they knew they had to have done, there's a certain process like this, if you need help we're here to help you. (Participant 11) |
| Providing continuous help to alumni | - I think university should be like a continuous help to you in terms of like, helping you know what's the next step, like okay so, for the people that recently graduated we had talks to maybe this country the specific hospital there for those that want to go for postgraduate internship there, you know, their options, maybe, if you want to do a master’s, this is what's required of you in this country, China or, then the other country. (Participant 11) - The school should make a person, I mean, the alumni aware of more of the specialty programmes out there, like residency programmes, things like that. So that's what I feel. Yeah. And give more like, also like approach to how to get into the specialty programmes, things like that. (Participant 14) |
| Managing the finance | - I have to find other options or other means of finding money, which I don’t have, I’m only a student right now. So, I cannot move to my next stage until I find other means of raising some money or after working. (Participant 15) |
| Improving one’s competence | - I just have to keep on adding, every day, keep on improving, that's progress yeah. (Participant 15) |
| Communicating with family to reach a consensus | - I want my family and this thing, like I have already like spoken with them, and discussed with them, and told them my plans and everything, and tell them to support me, because their support can help me. (Participant 06) |
| Providing help with issues related to the COVID pandemic | - Yeah, internship to start and it delayed graduation. That is one important factor to be considered. And they would like to know if there are any internship options that are from our school that are available in other countries. (Participant 08) |
| Providing the needed support related to overseas medical education | - I feel like the school should implement a programme that helps students study for potential about the exams, I know they can't really focus on anyone, they could focus on a general exam, kind of scenario, which will help students directly (Participant 17) |
| Making adjustments to curriculum and extra-curriculum activities | - I'm not saying it this way, I need more experience, even for being a general practitioner, we need to double our effort than the domestically-trained medical students. But even after that big effort, there will be areas we want to be and not be able to cover because we are not able to get the fact. So, I'm really hoping we can all aspects. (Participant 18) - Now, if they do something like that from year one, by year six the school will have many publications from undergraduates. (Participant 05) |
| Thinking logically and sorting out the difficulties | - I try to decide to do my own plans. Every time, like I expand my choices. I try to accomplish something, and another challenge and keep on. Yeah, keep on going like that. (Participant 02) - I think a timeline to help me, like you know, about last day or something like that could really help me make my decision. (Participant 12) |
| Standing firm for one’s original aspiration | - I feel like once I’ve made up my mind, that put myself into it, I feel I am capable of finishing it, it’s what I paid, no matter how difficult or hard it is, so, for me it is all about just making up my mind and getting to the point of the beginning. (Participant 12) - And later you do the research, prepare and self research or what you want to do and what you want to pursue in career thing. And just stick on that. (Participant 08) |
| Eliminating the unlikely options | - So, I think one thing I know I struggle with is dealing with being on the computer and reading and such jobs, so I know such things as radiology are not for me, medical law is not for me, epidemiology such public medicine, I’ve already cut them out, because I know they're not my strength, they're not where my strength is. I love being hands-on, so I’d rather be an emergency doctor, I’d rather be a surgeon, I’d rather be a dermatologist, because I also get to interact with a patient, on a personal level instead of on the computer. (Participant 07) |
| Confirming the indecisiveness | - And in terms of knowing whether it is actually what I want to do. I do like some psychology courses on the side as well. So that, and I noticed that, oh I actually do enjoy this. So I get reassured in myself that this is the right direction. Yeah. (Participant 01) |
| Teaching effective career decision making skills | - I think it can have some counsellors that we can talk to, about our career goals or specialisations. It would be really great for people to actually know what's involved. The challenges, the advantages and disadvantages involved in the path you have chosen. (Participant 15) |
| Increasing self-motivation | - It should all be inside, you should be your best cheerleader at the end of the day. (Participant 05) |
| Reminding oneself about the inevitable struggles in advance | - My strategy is that career is not so easy, and no one can build their career without struggling. I have to struggle in my way. And I am ready to struggle. (Participant 10) - The profession which I’ve chosen, like the day one itself, I realized it’s not going to be easy, but I’ve taken it as a challenge. (Participant 13) |
| Setting a target to push oneself forward | - But I think I needed it (the idea of doing licensing exams) to give me a drive to read all these books, to do this research, to do everything I’ve done, and if I didn't have it, I’ll stop at doing exams and then forgetting everything. (Participant 05) |
| Raising students’ awareness of career planning and study planning | - I would recommend the university to help international students with, it would be this: It would be pushing forward the whole narrative of, guys, I think you need to add something on to whatever you’re trying to aim at, which is way further than graduation, so that it can pull you in front. (Participant 05) - You know, because you’re in China and it’s like, maybe I just focused on just doing what’s here and you’re not really focusing on what's next, you know, very few people focus on what’s next, because they think, oh six years I’ve got time. But once you in first year imagine if we got to first year, and we were taught okay you guys are in first year, but yeah a couple of things that you need to know if you intend to go to the USA at some point, you should start preparing for USMLE from your first year, up until you get to maybe fourth year first semester, you'll be ready, either the resources that are available to you, the books like this, and the library, your seniors can help you. (Participant 11) |
| Thinking positively and being unafraid to make mistakes | - The second thing you have to be unafraid, unafraid to make mistakes. You have to understand that you will make mistakes, and you will make the wrong decision. And you have to be able to understand that, even if I make the wrong decision, there is also chance to make it right. (Participant 17) - I wouldn’t say I’m looking at it in a way that’s affected me really negatively like as if it’s totally tearing my life apart, so I prefer to look at it as a positive. So it has not really influenced my future career plans. (Participant 05) |
| Seeking comfort from outside | - Sometimes I just, I talk to my friends. And they just tell me, oh you worry too much, you know. You shouldn’t be thinking like that, blah blah. (Participant 11) - First of all, I'm a Christian, I believe in God. So firstly, I pray to God about all the problems that I encounter and everything I have. (Participant 15) |
| Leaving it for now and revisiting later | - I just clear my mind, you know, put away everything relating to that career. Maybe go out, just go out, go for a walk. Just basically clear my mind and just do other things that distract myself, distract myself from things that relating to my career decision or what things of that nature, just keep the sets of mind, you know, not bother too much about it. Then revisit those difficulties I'm facing. (Participant 14) - I am just one person who can, I’ll just put it on hold, I’ll put the thought on hold, and maybe just tried to do other things, and say okay, I will return to this task, a bit later when I feel like I’m a bit more settled, you know, to make this decision. (Participant 11) |
| Doing physical exercises to relax | - And when I feel depressed, I will workout. (Participant 13) |
| Having a flexible timeframe | - I’ll be like, I want to be so and so in my life, that would maybe take me two years or five years, or maybe 10 years, but I would still be happy that I have reached that point. (Participant 13) |
| Having a backup plan | - And still if I work hard and still, I don't get, then I’ll give it a second chance, maybe after one year. Yeah, I’ll try to give another chance. If still after a second chance, also nothing happens, then I’ll try to directly open the hospital. So that if one or another, I have to have some backup plans. (Participant 06) |
| Providing service related to mental health | - Talk to students about the mental health. (Participant 04) |
